# The Impact of Using Digital Video Recordings by Prospective Teachers on Their Technological Pedagogical Content Knowledge

**DOI:** 10.3390/ejihpe14090162

**Published:** 2024-08-25

**Authors:** Shaheen Shayeb, Wajeeh Daher

**Affiliations:** Faculty of Graduate Studies, An-Najah National University, Nablus P400, Palestine; shahen.gzali@gmail.com

**Keywords:** digital video recordings (DVRs), prospective teacher, technological pedagogical content knowledge (TPACK)

## Abstract

Analyzing digital video recordings (DVRs) is an effective instructional strategy for professionally preparing and developing prospective teachers. This study examines the impact of using DVRs among prospective teachers at Al-Qasimi College on their technological pedagogical content knowledge (TPACK) and its seven domains (TK, PK, CK, PCK, TCK, TPK, TPACK). The study was based on a mixed methodology approach, and the study sample included 70 prospective teachers who were distributed into an experimental group and a control group. Data were collected using the TPACK questionnaire and semi-structured interviews with 10 participants. The quantitative and qualitative results showed that prospective teachers in the experimental group significantly and positively impacted TPACK and its seven domains. The results of the study indicated that digital video recordings allowed prospective teachers to reflect and ponder on their teaching skills, content delivery, and the appropriate use of technology and its relevant tools in the educational process to identify strengths for development and weaknesses for improvement. Therefore, the results of the current study provide concrete evidence for the need to use DVRs as a promising educational approach in future professional preparation and to develop prospective teachers’ technological pedagogical content knowledge (TPACK).

## 1. Introduction

The primary goal of teacher education programs is to equip future educators with a strong skill set for effective teaching. This includes fostering a growth of a mindset that encourages continuous development, utilizing technology for pedagogical purposes, and ultimately creating an environment conducive to student learning success. This environment could entail teachers’ integration of ICT in a community of practice [1], technology integration as an innovative practice [2], and the design of activities by prospective teachers in STEM contexts [3]. However, a documented phenomenon known as “practical practice shock” highlights the gap between theoretical knowledge acquired in teacher preparation programs and its real-world application [4]. This underscores the need for pedagogical interventions that bridge the theory–practice divide [5]. Research suggests that fostering a contextually grounded learning environment is critical in this regard [6,7]. In essence, making practice readily observable for prospective teachers facilitates effective learning and promotes a holistic, integrated approach as opposed to fragmented experiences [8,9,10].

Digital video recordings (DVRs) have emerged as a promising tool for promoting reflection and self-analysis among prospective teachers [11]. These recordings enable prospective educators to meticulously analyze classroom events that might escape immediate attention, while simultaneously providing rich data for self-evaluation and learning from past experiences [12,13].

The Technological Pedagogical and Content Knowledge (TPACK) framework is a professional development program [14]. It offers a structured approach to identifying the knowledge domains prospective teachers need to acquire to effectively integrate technology within their pedagogical practices [15,16]. This study employed the TPACK framework as both a conceptual foundation and an analytical tool for exploring prospective teacher knowledge within the context of a digital recording environment.

## 2. Literature Review

### 2.1. Preparing Prospective Teachers

Digital technology is suggested as a tool in educational settings, where it can assist students and teachers in the different educational aspects. Some of the researchers were concerned with the impact of technology on students’ learning. Mater et al. [17] reported that the STEAM environment strengthened the mental motivation of middle school students. Salhab and Daher [18] reported that university students’ engagement increases as a result of their involvement with mobile learning. Some researchers were concerned with the impact of technology on teachers’ instruction. Najjar and Daher [19] reported the positive impact of a training program based on a technology-rich program on instruction concerned with scientific inquiry. Some of the researchers were concerned with the role of the Ministry of Education in the integration of technology in their institutions. Daher and Salameh studied the role of the Ministry of Education in addressing distance education during emergency education [20].

The role of technology in educational settings is especially true for video and videoing [21,22]. In general, current teacher preparation programs within colleges typically prioritize face-to-face instruction and mentorship [23]. This traditional, top-down approach focuses on verbal feedback to rectify observed teaching deficiencies [24]. However, this methodology offers limited opportunities for prospective teachers to critically examine their diverse teaching skill sets [25,26]. To address this, alternative approaches have emerged that emphasize fostering a deeper understanding of pedagogy through dialogue, reflection, and self-analysis [27]. Technological advancements, particularly in educational technology, offer a suite of tools to enhance self-reflection and analysis of teaching situations, ultimately leading to improved teaching practices. Examples include the use of digital video recording (DVR) technologies for self-reflection and analysis [28].

### 2.2. Digital Video Recordings (DVRs) Enhance Prospective Teacher Preparation

The use of digital video recordings (DVRs) is based on the constructivist learning theory, in which learners play active and effective roles [29], learning best when they discover things themselves and take control of their learning [30].

DVRs are an effective tool for promoting reflection, thinking, and self-evaluation among prospective teachers [11] due to their unique ability to provide a rich and complex view of teaching practices and reflect on them, analyzing them to identify strengths and weaknesses in performance [31,32]. This allows for more informed decisions about future teaching and learning in the classroom, and the additional review and reflection process on prospective teachers’ classroom teaching practices can lead to an increase in the amount of experience [33]. Several studies have shown that the use of DVRs promotes reflection and critical thinking in video clips, enabling prospective teachers to self-analyze their strengths and weaknesses for development and improvement [34,35]. While some studies have reported no significant differences in the reflections of teachers who used video editing to support their written reflections and those who wrote reflections without video editing [36].

The combination of reflection on practice and effective use of video can enhance professional development for teachers [37]. Several studies have highlighted the impact of video on the development of various teaching skills among teachers. Teachers who use video with evidence-based reflections instead of memory become able to uncover their implicit beliefs and gain a deeper understanding of what they teach, demonstrating teaching effectiveness [38]. While a study by Guo found that video data provide avenues for prospective teachers to better self-evaluate, this approach has helped develop their teaching strategies and build their teaching confidence through practice and reflection [39]. A study by Snoeyink revealed that self-video analysis enhanced prospective teachers’ skills in several areas, including observing all aspects of lessons, developing classroom management skills, understanding individual student needs and content comprehension, and improving their teaching practices [40].

Teachers reported that self-video analysis encouraged them to change and helped them in several areas, including seeing their teaching from a new perspective, feeling responsible for changing their practices, and seeing their progress. This led to a positive change in future teaching practices [41]. The comments embedded in the video helped prospective teachers better understand their performance and identify strengths and weaknesses, thus aiding in professional development and improving their teaching performance [42]. Data analysis in a study by Eltahir et al. shows that the experimental group that used the video recording tool was more effective in mastering specific teaching skills and showed higher motivation compared to the control group of students who did not use video recording activities [43].

The previous description of DVRs shows that for these recordings to succeed some conditions should be met. First, these recordings should be reflected upon by in-service or prospective teachers. Reflection is what makes DVRs vital for teachers’ development in their teaching. Second, the reflection can be undertaken individually by the individual teacher or by a group of teachers, where each of the two types of reflection supports the professional development of the teacher. Third, the participants in the DVRs should focus on change; i.e., their goal of watching and analyzing the DVRs should be the observation of weaknesses in their teaching and how to overcome these weaknesses.

### 2.3. The TPACK Framework as a Conceptual Framework

In this study, the Technological Pedagogical Content Knowledge (TPACK) framework was used as a conceptual framework. It was based on the pioneering Pedagogical Content Knowledge (PCK) framework developed by Shulman [44]. Pierson, Mishra, and Koehler developed a model for integrating technology called Technological Pedagogical Content Knowledge (TPACK) [37,38]. They believed that Shulman’s Pedagogical Content Knowledge (PCK) framework was incomplete and hypothesized the need for a third knowledge domain, Technological Knowledge (TK), to prepare teachers for effective teaching in the 21st century [45,46].

**Figure 1:**
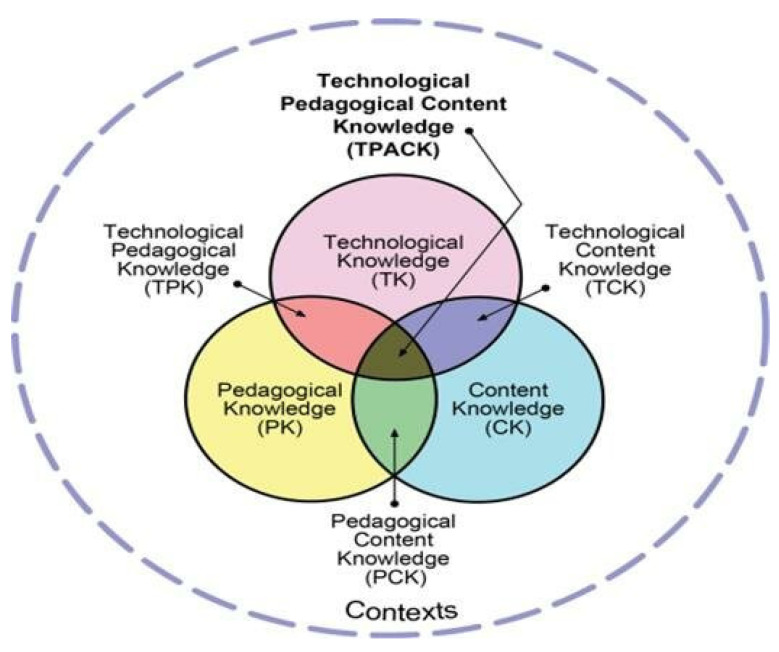
TPACK framework (reproduced by permission of the publisher, © 2012 by Mishra and Koehler [46].

This framework, in Figure 1, consists of seven knowledge domains: Technological Knowledge (TK), Pedagogical Knowledge (PK), Content Knowledge (CK), Technological Pedagogical Knowledge (TPK), Technological Content Knowledge (TCK), and Pedagogical Content Knowledge (PCK). The integration and interaction of these seven knowledge domains is known as Technological Pedagogical Content Knowledge (TPACK) [47].

### 2.4. Using DVRs to Analyze TPACK Domains

Teacher preparation focuses on improving and developing diverse knowledge in all TPACK domains, enabling teachers to manage the classroom in a technologically enhanced environment. Researchers believe that teacher preparation programs should provide opportunities for prospective teachers, not only to develop their knowledge but also to practice it in the classroom [48]. The use of DVRs has emerged as a viable solution to help prospective teachers develop TPACK.

Two studies [49,50] showed a significant positive impact of using DVRs on the development of TPACK domains, except for content knowledge (CK), which was lower. A study by Fan and Salleh indicated a significant improvement in students’ conceptual understanding of the concept of respiration for the experimental group after the intervention of embedding video technology [51]. Nilsson and Karlsson [52] suggested that video feedback can be useful in supporting the development of pedagogical content knowledge (PCK). Jang [53] showed that the use of DVRs had a significant impact on all TPACK domains except for technological knowledge (TK). Karlsson and Nilsson [54] concluded that self-recorded video clips with written comments helped to show aspects of their knowledge within the TPACK framework. Forsler et al. [55] showed that teachers were able to identify challenges they faced in teaching the topic of sustainable development (RCM) through video-based reflection and were able to develop new strategies for teaching the topic of sustainable development (RCM) through video-based reflection.

### 2.5. Study Rationale, Objectives, and Questions

Prospective teachers face a gap between what they learn from educational theories and learning within their specialization, and practical practices in the classroom. These practices rely heavily on traditional face-to-face methods, with a focus on narrative in delivering information without a link to document these practices for the evaluation and guidance of the prospective teacher.

Based on the gap, using DVRs as a tool to document what happens in the classroom in different educational situations deepens the ability to reflect, observe, and analyze teaching from its different aspects [56]. This makes DVRs a possible tool to bridge the gap. A framework was needed to organize the knowledge of the prospective teacher to reflect on and analyze their lessons using DVRs [15,16]. Therefore, TPACK was employed as a conceptual framework for this study and an organizer of prospective teacher knowledge.

In light of the foregoing, this study aims to examine whether the use of DVRs by prospective teachers affects their Technological Pedagogical Content Knowledge (TPACK). The current study aims to answer the following research questions:

1. What is the impact of prospective teachers’ use of DVRs on their Technological Pedagogical Content Knowledge (TPACK) and its seven domains: (TK, PK, CK, PCK, TCK, TPK, TPACK)?
2. How does the use of DVRs affect Technological Pedagogical Content Knowledge (TPACK) and its seven domains: (TK, PK, CK, PCK, TCK, TPK, TPACK)?

## 3. Materials and Methods

### 3.1. Research Design

The study employed a mixed methods approach, combining both quantitative and qualitative methods to gain a deeper understanding of the quantitative data on the impact of DVRs on TPACK and its potential for generalization in different educational contexts [57,58].

For the quantitative strand, the researcher adopted a quasi-experimental design, utilizing a pre-survey and post-survey design with two groups: experimental and control. Both groups were administered a self-report questionnaire measuring the dependent variable TPACK before and after the intervention [59].

For the qualitative strand, a descriptive approach was employed for data collection and analysis. This approach involved describing the studied phenomenon as authentic as possible, through interviews with prospective teachers to examine their perceptions and experiences of their TPACK.

### 3.2. Participants

The study involved a sample of 70 prospective teachers at Al-Qasmi Academic College of Education. The college is in Baqa, Israel, and is located in a triangular area. It is populated by Arab Israelis. It teaches educational topics to prospective teachers in various disciplines such as Languages (Arabic, Hebrew, and English), science, mathematics, and computers, as well as early childhood and special education.

The participants were divided into two groups: experimental and control. The experimental group used DVRs in their lessons and reflected on and analyzed them. This group included nine male prospective teachers and 26 female prospective teachers. The control group did not use DVRs. This group included 11 male prospective teachers and 24 female prospective teachers. In addition, semi-structured interviews were conducted with 10 prospective teachers from the experimental group. Table 1 shows details of the interview participants.

### 3.3. Data Collection Tools

This section describes the quantitative and qualitative data collection tools, including the questionnaire and interview.

#### 3.3.1. TPACK Questionnaire

The present research utilized a self-report questionnaire for the TPACK domains and subdomains before and after the experiment [52], developed by Schmidt et al. [60]. The questionnaire consisted of 53 items in Hosseini and Kamal’s study [61]. This questionnaire aimed to assess the impact of prospective teachers’ use of DVRs on their TPACK. After translation and validation, the questionnaire contained 53 items, covering the seven TPACK domains: 11 items for (TK), such as “Using DVRs allows me to learn about technological tools and integrate them into teaching”; 7 items for (PK), for example, “DVRs enable me to learn how to organize and maintain classroom management”; 6 items for (CK), such as “DVRs help me gain sufficient knowledge about my specialty subjects”; 7 items for (PCK), for example, “DVRs make me capable of managing my students’ learning about a specific subject”; 5 items for (TCK), for instance, “DVRs allow me to learn about technological tools that I can use to teach a specific subject in my area of expertise”; 10 items for (TPK), such as “DVRs enable me to select technological tools that enhance my teaching methods”; and 7 items for (TPACK), for instance, “DVRs enable me to select technological tools that promote the learning of (a specific subject from my specialization) for a lesson”. A five-point Likert scale was used: (1) Strongly Disagree, (2) Disagree, (3) Unsure, (4) Agree, and (5) Strongly Agree.

The internal consistency of all items in the seven domains was verified, confirming the validity of the questionnaire item correlation coefficients, with a total internal consistency of (*p* = 0.001, P.C = 0.838). The overall reliability value of the questionnaire was 0.918, for the total 53 items. The reliability of the domains ranged from (0.886) as a minimum to (0.919) as a maximum (see Table 2).

#### 3.3.2. Semi-Structured Interviews

Qualitative data were collected through semi-structured interviews with the participating prospective teachers. The aim of these interviews was to explore their experiences using DVRs and the impact of this use on TPACK. The interview questions were based on the main research questions. An interview guide was developed to lead the interview. This guide included seven open-ended questions and probing questions. The aim of the interview was to complement the quantitative results, explaining and interpreting them. The participants agreed to have their interviews recorded via Zoom, and each interview took 30–40 min. An example of a central open-ended question and supporting sub-questions used in an interview: In the domain of TCK, how did analyzing DVRs help you select the appropriate technological tool to achieve student acquisition of the learning topic? A. Provide an example from a DVR you analyzed. B. How did you find it when the technological tool did not achieve subject acquisition or goal attainment? C. How did analyzing DVRs help you rectify the situation later?

### 3.4. Procedure

We chose the experimental and control groups from four specializations: mathematics, science, early childhood, and Arabic language, from the second and third years. The sampling was purposive. On the one hand, the participants represented the research population and, on the other hand, they expressed readiness for data collection. The first author explained the objectives and process of the research to all participating prospective teachers. The number of prospective teachers in each of the experimental and control groups was 35 prospective teachers. The experimental group used DVRs utilizing the IRIS CONNECT program to record lessons during the second semester of the 2022/2023 academic year. The students in the control group went through the preparation and qualification experience in a traditional way without using digital videoing, depending on traditional face-to-face meetings. Overall, the number of the quantitative study sample was 70 prospective teachers. Ten prospective teachers were selected purposively for the interview from the experimental group, where the selection was performed in coordination with the educational supervisors and based on the prospective teachers’ consent to participate in the interview.

The practical teaching experience method, using digital video recordings (DVRs) within the IRIS Connect program, was employed by the experimental group in the training schools. These DVRs, recorded on a mobile device using the IRIS Connect app and then uploaded to the IRIS Connect website, guided the reflection process of the prospective teachers. The prospective teachers shared their recorded lessons electronically, via the IRIS Connect website, with the pedagogical supervisor and fellow prospective teachers. IRIS Connect enables creating user accounts, adding these accounts in IRIS Connect, and pairing them with the recorded video. This allows electronic sharing, analysis, and feedback. Subsequently, the pedagogical supervisor and prospective teachers view the recorded lesson at their convenience (asynchronously). Three processes are then possible. The first entails the pedagogical supervisor who analyzes the entire recorded lesson, identifies strengths and weaknesses, and writes comments as feedback on specific teaching situations in the recorded lesson. The aim of these comments is to improve the gaps in the prospective teachers’ instruction, by relating to the TPACK framework. The second process entails peers. Peers can participate in analyzing the strengths and weaknesses of teaching situations. The third process entails the prospective teacher who can review her or his recorded lesson, identifying areas that did not relate to one of the TPACK components, according to the pedagogical supervisor and/or peers. Then, the prospective teacher responds to the comments. A plenary discussion takes place on the IRIS Connect platform to reach a consensus on the various observations with the goal of improving the teaching methods in future lessons. If the discussion is insufficient, the observations are discussed with the prospective teacher either face-to-face or synchronously via digital platforms such as Zoom.

The experimental group included 35 prospective teachers who applied to the program over two semesters of the academic year 2022–2023. They filmed their lessons completely or partially using DVRs, shared the recordings with their educational supervisor and colleagues, reflected on the recordings and analyzed the strengths and weaknesses of their lessons by rewatching the recordings, focusing on TPACK aspects, and then sought to improve weaknesses in future lessons, focusing on improving TPACK skills.

The control group included 35 prospective teachers who participated in traditional applications without using DVRs. Here, the supervision relies on the educational supervisor’s face-to-face observation of the lesson and colleagues, taking into account the pursuit of improving weaknesses, and developing strengths in future lessons.

### 3.5. Data Analysis

#### 3.5.1. Quantitative Data Analysis

Before performing the statistical tests, we examined the assumptions of these tests.

Assumptions of the Analysis of Covariance (ANCOVA): Assumption 1: Homogeneity of variance: Homogeneity was tested using Levene’s test. The test results show that there is homogeneity of variance in the following domains: TK, PK, and CK, as well as their statistical significance (*p* > 0.05). While the rest of the domains do not have homogeneity because their significance value is (*p* < 0.05). Assumption 2: Independence: The results show that there is no interaction between the dependent variable represented by the pre-TPACK questionnaire and the independent variable represented by the group type (control and experimental) because of the calculated significance level (*p* > 0.05). Assumption 3: Normality of data distribution in the control and experimental groups: The results of the Kolmogorov–Smirnov test show that all TPACK domains and the total scale do not follow the normal distribution, as the value of the statistical function in it was (*p* > 0.05). Based on the results of the previous assumptions, especially the results of the normal distribution, Quade’s Distribution-Free Test was used instead of ANCOVA for all TPACK domains as well as the total scale.

The assumptions of the Paired Samples *t*-Test: The assumption of a normal distribution for this test was examined in the pre- and post-questionnaires. The normal distribution results showed that all TPACK domains and the total scale were (*p* > 0.05). Based on this result, a paired-sample *t*-test was used for the pre-survey and post-survey.

The Quade’s Distribution-Free Test was used to answer the first quantitative question to compare the means of the experimental and control groups in the post-survey after controlling for the pre-survey scores that are not of primary interest in the study, as well as to show the effect of using DVRs in the seven TPACK domains and the total scale. In addition, this test is free of distribution, easy to use, and statistically strong [61].

The results of this alternative test (QUAD test) were written in accordance with the methodology described in related studies studies [62,63,64,65], such as Cangür et al. [62] and Stewart et al. [64], as well as the detailed instructions provided by Fauzi [65]. Instructions outline the theoretical and practical steps of the test using the SPSS 27.0 statistical software, including how to write and format the results tables.

The Paired Samples *t*-Test was used to compare the effect of DVRs within the experimental group before and after use in the seven TPACK domains and the total scale, and to compare the effect of using traditional methods to guide prospective teachers without using DVRs within the control group before and after the experiment.

#### 3.5.2. Qualitative Data Analysis

Using Thematic Analysis included six steps: Familiarization: Watch the interviews repeatedly until they become familiar. Coding: Assign codes to the interview data to facilitate its organization. Theme Development: Identify themes that align with the theoretical framework (TPACK) and its seven domains. Theme Review: Review the themes to ensure they accurately reflect the interview data. Theme Naming: Adopt the theme names from the theoretical framework (TPACK). Reporting: Write a results report that presents the identified themes [65]. Table 3 shows the categories, themes and examples on the themes that resulted from the interviews. 

The validity of the research procedure resulted from arriving at saturation, where saturation means that no new data, themes, or categories emerge when analyzing an interview transcript [66]. Despite our achieving saturation when analyzing the eighth interview, we continued the analysis of the ninth and tenth interview to make sure that all the themes had emerged.

## 4. Results

### 4.1. Quantitative TPACK Results

To answer the first research question regarding the impact of prospective teachers’ use of DVRs on their Technological Pedagogical Content Knowledge (TPACK) and its seven domains, we computed first the adjusted means and standard deviation of the TPACK components and the overall score for the experimental and control groups. Table 4 shows the results. 

Generally, the experimental group had higher mean scores in the TPACK components than the control group. To find the significance of this difference, we ran Quade’s test. Table 5 shows the results.

Table 5 shows that the differences in the mean scores of TPACK domains, and between the experimental and control groups, are significant.

In addition, to find whether there were significant differences in the domains of TPACK before and after the experiment for the experimental and control groups, we ran a paired sample *t*-test. Table 6 shows the results.

Table 6 shows that the participants in the experimental group improved significantly their perceptions of TPACK domains, while those in the control group did not. 

### 4.2. Qualitative TPACK Results

The second question, a qualitative one, was addressed through the analysis of interviews conducted with the prospective teachers. Thematic analysis was employed to analyze the data collected from the interviews based on the conceptual framework of the study, which is Technological Pedagogical Content Knowledge (TPACK) with its seven domains (TK, PK, CK, PCK, TCK, TPK, TPACK). The didactic supervisor presented excerpts from the prospective teachers’ statements (pseudonyms used) based on the categories grounded in the study’s conceptual framework.

#### 4.2.1. Technological Knowledge (TK)

The participants indicated that using self-reflection through DVRs helped them improve their technological knowledge (TK). Tasneem talked about her experience: “*I used PowerPoint in teaching the topic of fractional equations in middle school, but using the program was not beneficial*”. However, she replaced the program with another tool after reflection: “*When I noticed the gap in choosing the tool through the video, I replaced the program with another tool, which is GeoGebra, because it achieved the benefit*”.

It is evident from the participants’ perceptions that self-reflection through DVRs had a significant impact on (TK) in choosing the appropriate tool to achieve the desired benefit from using it.

#### 4.2.2. Pedagogical Knowledge (PK)

DVRs helped the participating prospective teachers improve and develop their teaching skills and pedagogical knowledge (PK). Joumana says: “*I noticed from the recording that the high-achieving students are sitting in the front, and they are the most participating in the topic (global warming), and some are sitting in the back. I did not care about their participation, focusing heavily on the students in the front of the class*”. However, she addressed the matter in later lessons: “*When I allowed the students in the back to participate in answering the review questions (global warming), I found that the students understood the topic, had answered the questions and have more information than others*”. Then, she concluded: “*It needs support and providing equal opportunities for everyone while considering individual differences*”.

It can be concluded that there is a positive and significant impact on the use of DVRs in pedagogical knowledge (PK) for improving and developing different teaching skills.

#### 4.2.3. Content Knowledge (CK)

The participating prospective teachers indicated that using DVRs helped them improve their content knowledge (CK). Suhad explained that the feedback from the educational supervisor through DVRs contributed to her guidance in dealing with the educational content: “*I passed the topic (near demonstrative pronouns) for the second primary grade, and I read the educational supervisor’s note, which indicated that there are some near demonstrative pronouns that I had studied and are not required in the curriculum for the second grades*”. The educational supervisor asked Suhad to review the curriculum. “*His guidance was in place due to the difficulty of teaching it to the second grade, so I addressed the matter the following week, and I deleted the unwanted demonstrative pronouns from the plan*”.

Shada said that one should not try to reach all methods to simplify any topic in mathematics: “*I looked at the curriculum, I did not see a detail of how to reach the laws (short multiplication), but when I searched for a tool to help me on how to reach these laws, and I presented it to the students, and filmed the lesson with a digital video, it became clear to me that we should not be satisfied with the mathematics curriculum to pass a specific topic, but rather look for other tools such as digital links and computer presenters (PowerPoint) and explanatory videos on the topic, in order to simplify the content and explain it in the required form*”.

It is noted that the impact of using DVRs is positive and significant in identifying errors in content knowledge to correct and evaluate them.

#### 4.2.4. Pedagogical Content Knowledge (PCK)

The participating prospective teachers reported that using DVRs helped prospective teachers reach effective teaching methods that enable students to acquire the content effectively. Maryam stated that changing teaching methods contributes to student interaction with the content: “*I initially taught the topic of reptiles in a traditional way, but I noticed that the students did not master the topic effectively*”. So, Maryam decided to plan the same lesson in a different educational way: “*I followed the group learning method in teaching the topic, which depends on research and exploration outside the classroom walls. I designed a picture for each reptile they found with a barcode, so that each group can enter a website or YouTube (YouTube) through the barcode (QR) to collect information about the reptile. I also built activities through the Genially website and designed a QR code for each activity. It was a great lesson, as the students interacted with the content in a way that indicates that they understood the topic as required*”.

It can be concluded that the impact of using DVRs helped prospective teachers identify and correct errors in their pedagogical content knowledge.

#### 4.2.5. Technological Content Knowledge (TCK)

The participating prospective teachers indicated that using DVRs played a significant role in helping them facilitate their technological content knowledge (TCK). Hassan said, “*If we wanted to bring the content we wanted to convey to the students to life, we would choose the technological tool carefully; so that the students could interact with it*”. Hassan provides an example of this: “*I found that with the Canva program, which allows the design of educational materials in an interactive way such as videos and interactive presentations, the students designed a comic strip for the seventh grade using Canva, and the students interacted with that, and the best thing was when each student shared what he designed with his colleagues*”.

It is noted that the use of DVRs had a significant and positive impact on the choice of the purposeful technological tool that makes students interact with the content in a more effective way.

#### 4.2.6. Technological Pedagogical Knowledge (TPK)

The participating prospective teachers indicated that using DVRs improves and develops technological pedagogical knowledge (TPK). Maha believes that the choice of the technological tool comes in order to choose a teaching method through which students interact with the educational content: “*I used the Word Wall application to teach the topic of relative pronouns for the second grade, and I found that this application needs to use the cooperative learning strategy so that the students can interact in their learning until they achieve the required goals*”.

As for Marwa, she saw that the assessment or summary of the lesson should be accompanied by a technological tool so that the students interact with the content they have acquired: “*When I wanted to assess the students’ knowledge in the topic of (extracting the common factor) for the seventh grade at the end of one of the lessons that I recorded digitally, I found that the students did not interact because the assessment was traditional and devoid of a technological tool, but I addressed the matter in the lesson that followed, where I used the Kahoot tool and also used the Word Wall application, by distributing the students into groups and sometimes in pairs in case the phones were not available”. Marwa continued by saying that the students showed interaction: “The students interacted with the content they had acquired, and they showed the required understanding and internalization of what they had learned*”.

Using DVRs made the prospective teacher choose the purposeful technological tool that makes their educational methods more effective, achieving meaningful learning.

#### 4.2.7. Technological Pedagogical Content Knowledge (TPACK)

The participating prospective teachers reported that using DVRs had a significant positive impact on the development of TPACK. Tasneem found: “*Repeating the reflection on my applied mathematics lessons from the video clips made me identify the mistakes I make*”. Tasneem’s result was: “*This made me use the technological tool that might be better than the tool I used previously and was not effective in my lesson, until I achieve my goals that make it easier for students to acquire the content in an active educational way and style*”.

DVRs allowed the prospective teacher to choose the most appropriate technological tool for educational content through teaching methods and strategies for the gradual elements of the content in order to achieve the goals.

## 5. Discussion

### 5.1. Impact on Pedagogical Content Knowledge (TPACK) Reflection

The study demonstrated that using DVRs as a professional development tool for prospective teachers had a significant impact on their TPACK, perceptions, and experiences. Analyzing video recordings of their lessons helped them reflect critically on their performance, identify strengths and weaknesses, and exchange experiences with supervisors and peers to develop their skills in integrating technology into teaching. This positive impact is attributed to guided reflection and peer dialogue in helping teachers connect theory to practice, improve their awareness of their practices, correct their mistakes, and focus their activities on student needs. This explanation is consistent with the findings of the present research, whether quantitative or qualitative, in addition to its consistency with previous studies [32,33].

The positive impact is explained by the integration of reflection and critical thinking through DVRs supported by the TPACK framework. This framework guided the prospective teacher’s assessment of their performance in various areas, becoming an analytical approach to their teaching practices. It also provided a framework for exploring knowledge, allowing for careful planning to improve and develop the next lesson [32,37]. This confirms that the combination of reflection and thinking in practice and the effective use of video can enhance the development of prospective teachers’ knowledge in its different domains [35]. While one study found no difference between prospective teachers’ reflections embedded in DVRs of lessons and written reflections without using DVRs [34].

### 5.2. Impact on Technological Knowledge (TK)

The present study’s quantitative findings showed the effective impact of prospective teachers using DVRs on TK. DVRs motivated participants to monitor, review, and evaluate their knowledge during their recorded lessons, which led participants to identify their strengths and weaknesses, describe ways to facilitate the development of this knowledge, and increase their awareness of the importance of strengthening it. They also learned how to use the technological tool correctly. This was undertaken by changing the technological tool if it was not effective in achieving purposeful integration, as presented in the interviews, which is in line with previous studies [58,59]. Prospective teachers used DVR analysis to improve their knowledge of integrating TK tools into future lessons. The results of the present research support previous studies that emphasized the value of self-video analysis for learning [51,53]. While one study confirmed that the impact of using digital video recordings on TPACK was less [52].

### 5.3. Impact on Pedagogical Knowledge (PK)

The quantitative and qualitative findings of the current study showed a positive impact on the use of DVRs in pedagogical knowledge (PK). This helped them to improve and develop (PK), which is explained by the practice of systematic reflection and thinking based on (TPACK) of recorded lessons that show their practices and teaching skills and the extent of their knowledge in this field (PK) and to identify the mistake made by the prospective teacher to share with his supervisor and colleagues to deal with the mistake and show the best way to deal with it [33]. Individual analysis of the video clips enabled the prospective teacher to identify his strengths and weaknesses in the recorded lessons to enhance the personal development of prospective teachers in the context of their pedagogical knowledge [25]. In addition, the interviewees reported that they were able, for example, to choose alternative teaching methods or consider individual differences, classroom participation, classroom management and discipline, and other teaching skills. This result is consistent with previous studies [42].

### 5.4. Impact on Content Knowledge (CK)

The quantitative findings of the study showed that the use of DVRs had a significant and positive impact on CK. This is due to teachers’ reflection on and analysis of lesson recordings, which helped them understand student interaction with the content and better guide their knowledge. This explanation is consistent with the qualitative results. The sense of responsibility for improving the content also motivated teachers to learn and develop [40]. This result is consistent with previous studies [53,54], while one study found in its results that there was a significant improvement in the student’s conceptual understanding of one of the concepts of the science subject (respiration) for the experimental group after the intervention using DVRs [44]. A study by Jang and Lei showed that self-video analysis had a less significant impact on CK [49].

### 5.5. Impact on Pedagogical Content Knowledge (PCK)

The quantitative findings of the study showed that the use of DVRs had a significant positive impact on PCK. This is due to teachers’ analysis of recorded lesson clips and their collaboration with colleagues and supervisors, as video data provide ways for prospective teachers to better evaluate themselves. This explanation is consistent with the qualitative results, as well as with previous studies [38]. This evaluation helped them develop their skills and identify effective strategies to improve student interaction with educational content. This result is consistent with previous studies [51,52,54], while a study by Jang and Lei indicated that the impact of using DVRs on PCK was moderate [49].

### 5.6. Impact on Technological Content Knowledge (TCK)

The quantitative results indicated that the use of DVRs played a significant role in helping them improve, develop, and facilitate TCK in a very positive way. This was positively reflected in the awareness and knowledge of TCK. This is consistent with the qualitative results where the prospective teachers said that the DVRs made them aware of the knowledge of TCK. It is also consistent with previous studies [67]. This result is explained by the fact that continuous reflection on recorded lessons enhances teachers’ skills in identifying strengths and weaknesses. A study by Fauziah et al. indicates that effectively integrating technology requires a deep understanding of how to use technology to deliver content effectively [68]. The choice of the appropriate technological tool must be consistent with the nature of the content and the needs of the learners [46]. 

### 5.7. Impact on Technological Pedagogical Knowledge (TPK)

The study’s quantitative results showed that the use of DVRs for analyzing recorded lesson clips, along with reflection and critique, helped prospective teachers enhance their TPK. The qualitative results showed that the DVRs not only helped enhance the TPK, but also helped develop it. By identifying strengths and weaknesses, the prospective teachers were able to integrate appropriate educational technology tools to improve their teaching approaches. This result can be explained by the fact that the integration and interaction between educational technology and pedagogical knowledge enabled the integration of technological tools to support the employment of various teaching methods, especially alternative methods that place the student at the center of the educational process. The prospective teacher’s awareness and knowledge of this connection and complementarity between technology and teaching made them ready to integrate educational technology that suits different teaching skills, such as the teaching method, the means of illustration, and others [48]. This can bring about a change in education due to the integration of purposeful educational technology. For example, a science teacher can employ the exploratory method (through trial-and-error learning theory) to examine a particular mineral through experimentation by using, for example, an augmented reality program. This result is consistent with previous studies that reported the positive influence of exploratory learning strategy [53,54].

### 5.8. Impact on Technological Pedagogical Content Knowledge (TPACK)

The quantitative results of the study showed that using digital video recordings (DVRs) to record and analyze lessons significantly improved prospective teachers’ TPACK. This is due to their increased awareness of the interaction and integration between the fields of technological knowledge, pedagogical knowledge, and content knowledge, as well as their ability to identify strengths and weaknesses in their teaching situations. This ability was present in the prospective teachers’ interviews. The improvement is attributed to prospective teachers becoming more aware of the role of technological tools in the educational process and how to use them effectively to enhance meaningful learning [69]. Being guided by the TPACK framework enhanced prospective teachers’ learning from their mistakes and development. This result is consistent with previous studies [52].

## 6. Conclusions and Recommendations

Agreeing with previous studies [70], the present study showed the positive impact video-based learning on students’ learning, here prospective teachers. The triangulation of the quantitative and qualitative results shows that the prospective teachers’ use of DVRs is an effective tool for reflection and contemplation on teaching, content, and technological tools during the educational process [28]. The study examines the impact of prospective teachers’ use of DVRs in TPACK areas.

The results of the study showed that the use of DVRs by teachers had a significant and positive impact on TPACK in favor of the experimental group. The prospective teachers’ perceptions were further supported and explained by the interviews. The interviews showed that the recordings allowed the prospective teachers to reflect and ponder on their teaching skills and content delivery. The quantitative and qualitative results indicate that the use of DVRs by prospective and in-service teachers is recommended, especially when the teachers collaborate and discuss the DVRs in a group. The discussion could also happen using tools such as the one used by the prospective teachers in their training. 

In addition, the research results showed that the appropriate use of technology and its relevant tools in the educational process could help enrich the teaching, and thus prospective, as well as in-service, teachers would use technological tools in their teaching, which would contribute to its improvement. 

The TPACK framework was used as a guide for the educational supervisor and the prospective teachers to monitor different educational situations to learn from the prospective teacher’s mistakes in teaching, content, and technology integration to bring about the desired change in future lessons. We recommend the adoption of the TPACK model to guide the education of prospective teachers and that there should be a guide based on the TPACK components to enable prospective teachers. Prospective teachers can reflect on and provide meaningful feedback on their educational processes related to all the components of the TPACK model.

## 7. Limitations of the Study

The main limitation was the inability to provide a unified control group and a unified experimental group, especially since practical training groups are small groups. The experimental and control groups were formed from different specializations as each specialization included a small number of prospective teachers. 

Another limitation is the self-reported responses on the questionnaire related to the TPACK domains. The generalization of the results should take into account the fact that the responses are self-reported. 

## 8. Ethical Procedures

The ethical standards of practical research were adhered to in all its stages and steps. The researchers sought official approval from the research authority, the academic dean at Al-Qasimi College, and the Practical Training Department to proceed with collecting data from the study sample; the approval for collecting both types of data was granted on 3-4-2023. The first researcher explained to the participants the nature of the study and its objectives through the questionnaire and interview and that the collected data would be kept confidential and would only be used for scientific and research purposes. Participation in the quantitative and qualitative study experience was optional, with the approval of the educational supervisor and the participating prospective teachers.

## Figures and Tables

**Table 1:** Details of the interview participants, using pseudonyms.

Pseudonym	Gender	Year	Specialization
Hassan	Male	Second	Arabic Language
Suhad	Female	Second	Arabic Language
Marwa	Female	Third	Mathematics
Shadha	Female	Third	Mathematics
Sahar	Female	Third	Mathematics
Tasneem	Female	Third	Mathematics
Huda	Female	Second	Early Childhood
Maha	Female	Second	Early Childhood
Jumana	Female	Third	Science
Mariam	Female	Third	Science

**Table 2:** Reliability using Cronbach’s Alpha, for the questionnaire, a domain of TPACK, and the overall questionnaire reliability.

	Number of Items	Reliability Using Cronbach’s Alpha
TK Domain	11	0.902
PK Domain	7	0.919
CK Domain	6	0.909
PCK Domain	7	0.905
TCK Domain	5	0.886
TPK Domain	10	0.894
TPACK Domain	7	0.895
Overall questionnaire reliability		0.838

**Table 3:** TPACK Interview Categories and Themes.

Category	Themes	Examples on Themes
TK	Knowledge and Use	I lacked proficiency in using the application effectively, particularly the technical aspects.
	Mastery	Once I gained proficiency in the application (Word Wall) and its settings, I was able to use it more effectively in another lesson.
	Suitability	I sought to replace the program with a tool that students could interact with, such as GeoGebra.
PK	Knowledge	I discovered through DVRs that this requires knowledge of how to support and provide equal opportunities for all while considering individual differences.
	Understanding	I saw through DVRs that the teaching method needed to be changed in the next lesson, so I used the cooperative learning strategy.
CK	Accessing	I found through DVRs that to expand the educational content, and students understand it better; We need to search for other sources.
	Mastery	The DVRs helped me to master the educational content so that I was able to repeat it in a way that makes it easier for students to understand.
	Addressing gaps	Through DVRs, it became clear to me that we should not rely solely on the math textbook to pass a particular topic.
PCK	Methods and means	Replacing the traditional teaching method with the “Think-Pair-Share” strategy led to improved student engagement and outcomes.
TCK	Selection	I noticed that the integration of technological tools and their selection should be developed for students to achieve their learning objectives.
	Interaction	I saw that the students interacted with the technological tools I chose, and these tools achieved learning and acquisition of the content.
TPK	Technology enhances teaching skills.	Using educational technology tools has made me more attentive to planning my lessons.
	Choosing the most suitable technology tool	I integrate the technology tool that achieves the educational objectives.
	Exposure to technology tools that play a role in teaching.	We should be exposed to new technology tools that are more effective in teaching and learning.
TPACK	Interaction	Recognizing traditional method’s limitations, I leveraged DVRs to explore a new engaging tech tool for a more active learning experience.

**Table 4:** Descriptive Statistics for Experimental and Control Groups on Post-Survey.

Domains	Group	N	Adjusted Means	S.D.
TK	Experimental	35	46.19	16.56
	Control	35	24.81	18.08
PK	Experimental	35	44.05	17.61
	Control	35	26.94	19.42
CK	Experimental	35	43.73	16.63
	Control	35	27.26	19.80
PCK	Experimental	35	44.31	16.83
	Control	35	26.68	19.28
TCK	Experimental	35	47.37	14.40
	Control	35	23.63	17.75
TPK	Experimental	35	47.13	16.42
	Control	35	23.86	16.85
TPACK	Experimental	35	46.96	16.21
	Control	35	24.03	17.25
Overall scale of the questionnaire	Experimental	35	47.56	15.19
	Control	35	23.43	17.31

**Table 5:** Quade’s Distribution-Free Test for the Effect of Video Use on TPACK.

Domains	Group	Sum of Squares	df	Mean Squares	F	*p*	$n^{2}$
TK	Group	6974.109	1	6974.10	23.144	0.001	0.254
	Error	20,490.877	68	301.33			
PK	Group	4214.537	1	4214.53	12.059	0.001	0.151
	Error	23,772.11	68	349.59			
CK	Group	4598.129	1	4598.12	13.882	0.001	0.170
	Error	22,523.83	68	331.23			
PCK	Group	4857.549	1	4857.54	14.707	0.001	0.178
	Error	22,460.60	68	330.29			
TCK	Group	9504.822	1	9504.82	36.356	0.001	0.348
	Error	17,777.62	68	261.43			
TPK	Group	9445.370	1	9445.37	34.101	0.001	0.334
	Error	18,834.69	68	276.98			
TPACK	Group	9174.916	1	9174.91	32.704	0.001	0.325
	Error	19,077.03	68	280.54			
Overall	Group	9683.887	1	9683.88	36.231	0.001	0.348
	Error	18,175.37	68	267.28			

**Table 6:** Pre- and post-survey mean, T-value, significance value, and effect size for the control and experimental groups of paired samples (Paired Samples *t*-Test).

Domains	Group	Type of Measurement	Mean	SD	t	*p*	d
TK	Control	Posttest	3.1766	0.88496	0.359	0.361	0.061
		Pretest	3.0909	0.88497			
	Experimental	Posttest	4.1039	0.59619	4.930	0.001	0.833
		Pretest	3.1766	0.88496			
PK	Control	Posttest	3.8245	0.74802	−0.064	0.475	0.059
		Pretest	3.8367	0.61214			
	Experimental	Posttest	4.4286	0.49000	6.531	0.001	1.104
		Pretest	3.6000	0.61912			
CK	Control	Posttest	3.8238	0.85934	−0.301	0.383	−0.051
		Pretest	3.8905	0.77115			
	Experimental	Posttest	4.4571	0.45615	6.187	0.001	1.046
		Pretest	3.5610	0.73224			
PCK	Control	Posttest	3.8000	0.86910	−0.288	0.387	−0.049
		Pretest	3.8612	0.66960			
	Experimental	Posttest	4.5061	0.46787	5.425	0.001	0.917
		Pretest	3.6137	0.75535			
TCK	Control	Posttest	3.3771	0.85924	0.382	0.352	0.065
		Pretest	3.2914	1.00977			
	Experimental	Posttest	4.2971	0.42391	5.480	0.001	0.926
		Pretest	3.3771	0.85924			
TPK	Control	Posttest	3.3486	0.92938	−0.740	0.232	−0.125
		Pretest	3.5200	0.83377			
	Experimental	Posttest	4.3114	0.53400	5.108	0.001	0.863
		Pretest	3.3486	0.92938			
TPACK	Control	Posttest	3.3265	0.90189	−0.859	0.198	−0.145
		Pretest	3.5306	0.86173			
	Experimental	Posttest	4.2980	0.55585	5.197	0.001	0.878
		Pretest	3.3486	0.92938			
Overall scale of the questionnaire	Control	Posttest	3.5253	0.68179	−0.265	0.396	−0.045
		Pretest	3.5745	0.66478			
	Experimental	Posttest	4.3432	0.41337	6.876	0.001	1.162
		Pretest	3.4291	0.62442			

## Data Availability

The data presented in this study are available upon request from the corresponding author. The data are not publicly available due to privacy concerns.
